# New insights into the regulation of Axin function in canonical Wnt signaling pathway

**DOI:** 10.1007/s13238-014-0019-2

**Published:** 2014-01-29

**Authors:** Xiaomin Song, Sheng Wang, Lin Li

**Affiliations:** State Key Laboratory of Molecular Biology, Institute of Biochemistry and Cell Biology, Shanghai Institutes for Biological Sciences, Chinese Academy of Sciences, Shanghai, 200031 China

**Keywords:** Wnt signaling, Axin, post-transcriptional modification, polymerization, auto-inhibition

## Abstract

The Wnt signaling pathway plays crucial roles during embryonic development, whose aberration is implicated in a variety of human cancers. Axin, a key component of canonical Wnt pathway, plays dual roles in modulating Wnt signaling: on one hand, Axin scaffolds the “β-catenin destruction complex” to promote β-catenin degradation and therefore inhibits the Wnt signal transduction; on the other hand, Axin interacts with LRP5/6 and facilitates the recruitment of GSK3 to the plasma membrane to promote LRP5/6 phosphorylation and Wnt signaling. The differential assemblies of Axin with these two distinct complexes have to be tightly controlled for appropriate transduction of the “on” or “off” Wnt signal. So far, there are multiple mechanisms revealed in the regulation of Axin activity, such as post-transcriptional modulation, homo/hetero-polymerization and auto-inhibition. These mechanisms may work cooperatively to modulate the function of Axin, thereby playing an important role in controlling the canonical Wnt signaling. In this review, we will focus on the recent progresses regarding the regulation of Axin function in canonical Wnt signaling.

## Introduction

Wnt signaling is one of the most conserved pathways during evolution, playing essential roles in multiple biological processes like embryonic development and adult tissue homeostasis. Dysregulation of the Wnt pathway underlies the pathogenesis of a variety of human cancers. So far, two types of Wnt signaling pathways have been characterized: canonical Wnt pathway (also known as Wnt/β-catenin pathway) and noncanonical Wnt pathway (including planar cell polarity pathway and Wnt/calcium pathway). The Wnt/β-catenin pathway is distinguished from the others in the way of its dependency on β-catenin, which is nevertheless not required for the two noncanonical Wnt pathways. As a central player in canonical Wnt signaling, β-catenin dictates the signaling intensity through its cellular levels and subcellular localization. In the absence of Wnt signaling, β-catenin is sequestered in the cytoplasm by the “destruction complex” composed of Axin, glycogen synthase kinase 3 (GSK3) and adenomatous polyposis coli (APC), etc. β-catenin is phosphorylated by GSK3 and subsequently subjected to degradation through the ubiquitin-proteasome pathway. Upon Wnt stimulation, low-density lipoprotein receptor-related proteins 5/6 (LRP5/6) is co-clustered with Frizzled (Fz) and Dishevelled (Dvl) and recruits the active Axin and GSK3 complex; these proteins thus form a high-molecular-weight assemblage close to the plasma membrane that is referred to as “LRP signalosome” (Bilic et al., 2007). Following the recruitment of Axin to LRP5/6, the “destruction complex” is disassembled; and β-catenin is released and translocates into the nucleus, where it forms a transcriptional complex with LEF-1/TCF and activates the transcription of Wnt-responsive genes. Recently, Li et al., based on their findings, proposed an alternative model that Wnt stimulation, rather than cause the disassembly of “destruction complex”, suppresses β-catenin degradation and leads to saturation of the complex by the accumulated β-catenin, thus sparing the newly synthesized β-catenin, which accumulates in a free cytosolic form and consequently enters the nucleus to bind LEF-1/TCF (Li et al., 2012).

Vertebrates have two Axin isoforms: Axin1 (also referred to as Axin) is constitutively expressed, while Axin2 (also called Conductin) is subjected to the regulation of Wnt signaling (Jho et al., 2002; Leung et al., 2002; Yan et al., 2001). Albeit different in their expression patterns, Axin and Axin2 appear to be functionally, at least partly, interchangeable in canonical Wnt pathway (Chia and Costantini, 2005). Considering its essential role in both “destruction complex” and “LRP signalosome”, the activity of Axin has to be tightly controlled. Post-translational modification is the most common mechanism for regulating protein activity. Several ways of modifications, such as phosphorylation, SUMOylation and ubiquitination, have been reported to play a role in regulating Axin stability, sub-cellular localization or association with other proteins (Callow et al., 2011; Fei et al., 2013; Kim et al., 2008; Kim and Jho, 2010; Zhang et al., 2011). Polymerization of Axin also regulates its activity in a way that currently isn’t clearly defined. Axin is considered as a protein that is largely unstructured except the N-terminal RGS domain responsible for binding APC and the C-terminal DIX domain for mediating oligomerization. Besides Axin, Dvl and Ccd1 also contain a DIX domain, which is responsible for their homo- and hetero- polymerization (Liu et al., 2011; Schwarz-Romond et al., 2007a; Shiomi et al., 2003). It is reported that DIX-mediated heterotypic interaction between Axin and Dvl may disrupt Axin’s association with the “destruction complex” and facilitate its recruitment to the “LRP signalosome” (Mendoza-Topaz et al., 2011; Schwarz-Romond et al., 2007b). Recently, two independent researches carried out by Sung-Eun Kim et al. and our group revealed a new mechanism of “auto-inhibition” behind Axin function, pointing out that conformational changes of Axin may play an important role in regulating its activity (Kim et al., 2013b; Wang et al., 2013). Notably, these mechanisms may be relevant and work cooperatively to fine-tune and regulate the function of Axin.

## Ubiquitination/Sumoylation of Axin

Ubiquitination is an important regulatory process for protein, playing diverse roles in modulating protein function. Ubiquitin is covalently attached to the targeted molecules through the sequential activities of three enzymes: ubiquitin-activating enzyme (E1) for ATP-dependent activation of ubiquitin (Ub), ubiquitin-conjugating enzyme (E2) for delivery of Ub, and ubiquitin ligase (E3) for ligation of Ub to the substrates. In this process, the E3 ligases are responsible for substrate selection and the specificity of Ub-linkage. There are seven lysines (K6, K11, K27, K29, K33, K48 and K63) in Ub molecule. Generally, proteins marked by K48-linked poly-Ub chains are destined to proteasome-dependent degradation, while other types of Ub chains could regulate a variety of cellular processes by both proteolytic and non-proteolytic functions (Kulathu and Komander, 2012). Multiple key components in canonical Wnt pathway are regulated by ubiquitination, such as β-catenin, APC and Dvl (Chan et al., 2006; Choi et al., 2004; Ding et al., 2013; Hu et al., 2010; Liu et al., 1999; Liu et al., 2001; Tran et al., 2013; Tran and Polakis, 2012; Wei et al., 2012; Winston et al., 1999). Despite limited reports so far on ubiquitination of Axin, it’s increasingly clear that Axin is also subjected to ubiquitin modification. Previously, Stegmeier lab revealed that Axin stability is regulated by Tankyrase and Tankyrase-mediated poly-ADP-ribose modification (PARsylation) of Axin is linked to Axin polyubiquitylation and subsequent degradation by the proteasome (Huang et al., 2009). Later, RNF146 was uncovered to be the E3 ligase for mediating Tankyrase-dependent degradation of Axin, thus playing a positive role in Wnt signaling (Callow et al., 2011; Zhang et al., 2011). Ubiquitin-specific protease 34 (USP34) is also reported to associate with Axin and control its levels, whereby modulating Wnt signaling (Lui et al., 2011). Smurf2 (Smad ubiquitination regulatory factor 2), a C2-WW-HECT type ubiquitin ligase, is suggested to be another E3 ligase for Axin, which also plays a role in regulating its stability (Kim and Jho, 2010). Interestingly, we recently uncovered that Smurf1, an isoform of Smurf2, also targets Axin, but for a non-proteolytic purpose. Our data suggested that Smurf1 ubiquitinates Axin through Lys (K) 29-linked poly-ubiquitin chains, which disrupts its interaction with LRP5/6 rather than leads to its degradation (Fei et al., 2013). Similar to Ubiquitination, SUMO modification of proteins also plays various roles in regulating protein function. However, researches regarding Axin SUMOylation are, so far, very limited. It was reported earlier that SUMOylation of Axin may regulate its function in noncanonical Wnt pathway (Rui et al., 2002). Recently, Kim et al. suggested that SUMOylation may protect Axin from ubiquitination, thus playing a role in regulating Axin stability in canonical Wnt pathway (Kim et al., 2008).

## Phosphorylation/Dephosphorylation Of Axin

Phosphorylation/Dephosphorylation is the most common post-translational modification for regulating protein function. Multiple components of Wnt pathway, such as LRP5/6, Dvl, β-catenin, TCF, are subject to reversible phosphorylation by kinases and phosphatases networks. Axin, as a crucial component of canonical Wnt pathway, is also under the regulation of phosphorylation/dephosphorylation. Without Wnt stimulation, Axin is phosphorylated, which enhances its binding affinity with β-catenin, leading to stabilization of Axin (Jho et al., 1999; Yamamoto et al., 1999); upon Wnt stimulation, Axin is dephosphorylated, leading to a less effective binding with β-catenin and consequently Axin degradation (Strovel et al., 2000; Willert et al., 1999). GSK3 is most likely the key kinase that phosphorylates Axin and facilitates its function in “destruction complex”. Besides GSK3, CKI was also observed to phosphorylate Axin both *in vitro* and *in vivo* (Gao et al., 2002). On the other hand, it is found that PP2A and PP2C may be the two phosphatases targeting Axin (Strovel et al., 2000; Willert et al., 1999). Recently, it was reported that phosphorylation of Axin by CKI may also improve its association with GSK3 and leads to a more active “destruction complex”, while protein phosphatase 1 (PP1) interacts with, and dephosphorylates Axin, reversing the effect conferred by CKI and hence contributing to the activation of Wnt signaling (Luo et al., 2007). Later, Jiang et al. reported that Dab2 could block the interaction between Axin and PP1, thus inhibiting Axin dephosphorylation and ultimately leading to inhibition of the Wnt signaling (Jiang et al., 2009). Previously, phosphorylation of Axin by Cyclin A/CDK2 was reported to increase its association with β-catenin (Kim et al., 2004). Recently, Axin was found to be phosphorylated by CDK5, and this phosphorylation facilitates its interaction with GSK3, which plays an essential role for axon development (Fang et al., 2011).

## Homo- And Hetero-Polymerization of Axin

**Figure 1:**
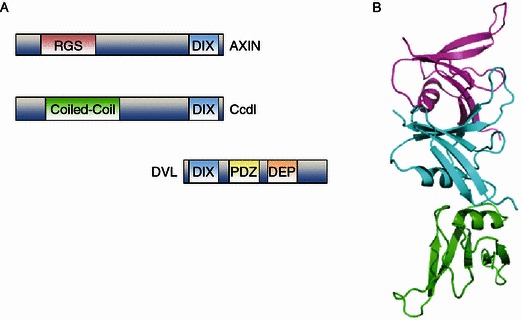
**The C-terminal DIX domain of Axin mediates its homo/hetero- polyermerization.** Schematic illustration of the domain organization for Axin, Dvl and Ccd1. These three proteins all contain a DIX domain, which mediates their homo- and hetero-interaction. The self-assembly of Axin-DIX or heterotypic interaction with Dvl-DIX may regulate Axin function in canonical Wnt signaling. (B) The three-dimensional structure of Axin DIX domain (PDB code: 1wsp) showed that it forms filaments in the crystal through head-to-tail self-interaction

It is not unusual that proteins modulate their functions through altering their oligomeric states or forming hetero-oligomer with other proteins. Self- or hetero-assembly of proteins also plays important roles in regulating Wnt signaling. For example, aggregation of LRP5/6 is essential for Wnt signaling activation, which may also require the oligomerization of Dvl (Metcalfe et al., 2010). Recently, we identified Caprin-2 as a new component of canonical Wnt signaling through facilitating LRP5/6 phosphorylation (Ding et al., 2008), and we further found that the oligomerization of Caprin-2 is required for its function in Wnt signaling (unpublished data). Axin contains a DIX domain of ~80 amino acids located at its C-terminus for mediating homo- or hetero-interaction, which seems to be essential for its function (Choi et al., 2010; Fiedler et al., 2011; Sakanaka and Williams, 1999; Yokoyama et al., 2012). The three-dimensional structure of Axin DIX domain showed that it forms filaments in the crystal through head-to-tail self-interaction (Schwarz-Romond et al., 2007a; Shibata et al., 2007) (Fig. 1B). Besides Axin, the homologous DIX domain is also present at the N-terminus of Dvl and the C-terminus of Ccd1 (also called DIXdc1) (Fig. 1A). It is argued that Dvl may release Axin from the “destruction complex” and deliver Axin to the “LRP signalosome” through DIX-mediated heterotypic interactions between Dvl and Axin (Schwarz-Romond et al., 2007b). Fiedler et al.’s recent work also indicated that Dvl may behave as a dominant-negative modulator of Axin to regulate its function via heterotypic interactions between their DIX domains (Choi et al., 2010; Fiedler et al., 2011). Ccd1 was initially identified as a positive regulator in Wnt signaling (Shiomi et al., 2003). Ccd1-DIX interacts with Dvl-DIX directly, converting latent polymeric Dvl to a biologically active oligomer(s) (Liu et al., 2011; Shiomi et al., 2003). However, the interaction between Ccd1 and Axin does not seem to be dominated by their DIX domains and other domains may also play an important role for their interaction (Wong et al., 2004).

## Auto-Inhibition of Axin

**Figure 2:**
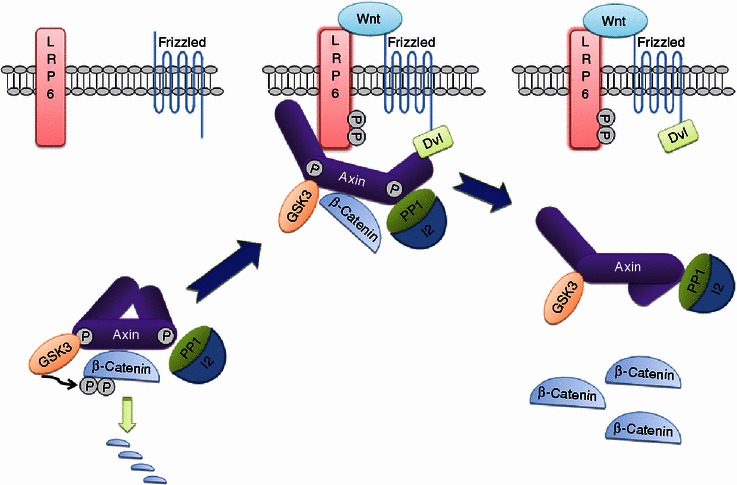
**The model of Axin autoinhibition.** Based on the findings of Kim et al. and us, we proposed a possible model for Axin “autoinhibiton”: without Wnt stimulation, the N-terminus of Axin interacts with its C-terminus, forming “type I” closed conformation, which blocks its binding with LRP5/6 but spares the binding site for β-catenin; upon Wnt stimulation, this type I auto-inhibited state of Axin is released for binding to LRP5/6 and the latter was subsequently phosphorylated, which inhibits the activity of GSK3 and thus moves Axin towards to the dephosphorylated state conferred by PP1. Dephosphorylated Axin forms a “type II” closed conformation through an intramolecular interaction between the C-terminus and the β-catenin binding region, making it unable to bind both of LRP5/6 and β-catenin

Conformational change is an important way for proteins to regulate their functions. In many cases, when a protein adopts an “auto-inhibited” conformation, its active site for substrates, or binding sites for other partners may be blocked and thus this state is also referred to as an “inactive” state; while, in an “active” state, the autoinhibitory conformation is released, making the active or binding sites available for interaction with substrates or regulatory partners. Normally, this reversible process of conformational change is induced and regulated by post-translational modifications or interactions with other proteins. Previous studies have suggested that Axin N-terminus (including the RGS domain and the linker region between the RGS domain and the GSK binding domain) plays an inhibitory role in Axin’s binding with its partners (Chen et al., 2009; Mao et al., 2001). However, the mechanism underlying such inhibition remains elusive. In our recent work, we revealed that the N-terminus of Axin is able to associate with its C-terminus, forming a “closed” conformation, which could be disrupted effectively by a small molecular compound, named as HLY78. HLY78 is an activator of Wnt/β-catenin signaling pathway identified by our group through high-throughput screening, which acts in a Wnt ligand-dependent manner (Wang et al., 2013). Our findings suggested that depending on distinct partners and post-translational modifications, Axin may undergo conformational change during its differential assemblies with “destruction complex” or “Wnt-LRP5/6 signalosome”. We further found that HLY78 targets Axin-DIX and triggers the conformational switch of Axin from a “closed” auto-inhibitory state to an “open” active state, leading to an enhanced association of Axin with LRP6 and subsequent activation of LRP6. Meanwhile, an independent work carried out by another group also proposed an “auto-inhibition” model of Axin (Kim et al., 2013b). In their model, phosphorylation by GSK3 makes Axin adopt an “open” conformation for binding to β-catenin or phospho-LRP6, while dephosphorylation by PP1 leads to a “closed” conformation (auto-inhibition) of Axin, making Axin incompetent for interacting with either β-catenin or phospho-LRP6. Notably, according to their findings, the C-terminus of Axin forms a “closed” conformation through an intramolecular interaction with the middle β-catenin binding region rather than the N-terminal region as we found. In our view, these two models are not in conflict with each other and may function alternatively or collaboratively in different cellular contexts. A possible model that accommodates both sets of findings is proposed in Fig. 2: without Wnt stimulation, the N-terminus of Axin interacts with its C-terminus, forming “type I” closed conformation, which blocks its binding with LRP5/6 but spares the one with β-catenin; upon Wnt stimulation, this type I auto-inhibited state of Axin is released for binding to LRP5/6 and the latter was subsequently phosphorylated, which inhibits the activity of GSK3 and thus moves Axin towards to the dephosphorylated state conferred by PP1. Dephosphorylated Axin forms a “type II” closed conformation through an intramolecular interaction between the C-terminus and the β-catenin binding region, making it unable to bind both of LRP5/6 and β-catenin.

## Micrornas Mediated Regulation of Axin

MicroRNA (miRNA) is a small noncoding RNA molecule normally containing 19–25 nucleotides in length, which regulates gene expression both at transcriptional and post-transcriptional level. So far, thousands of transcripts have been reported to subject to microRNA regulation, playing extensive and important roles in eukaryotic development and physiology. Moreover, increasing evidences have implicated the role of miRNA deregulation in human disease including cancer. Recently, there are several researches addressing potential roles of miRNAs in Axin regulation. In a screen for miRNAs affecting Wnt pathway, Silver et al. found that ectopic miR-315 directly inhibits Axin as well as Notum (Silver et al., 2007), thus acting as an activator of Wnt signaling. Later, another miRNA—hsa-miR-34a—reportedly targets both the 5′-UTR and 3′-UTR of *Axin2,* whereby repressing *Axin2* expression (Lee et al., 2009). Recently, Egea et al. revealed that *lethal-7 (let-7)* also targets and represses the translation of *Axin2*. *lethal-7 (let-7)* is one of the first two known miRNAs (the other one is *lin-4*) (Reinhart et al., 2000), which is highly conserved across species and implicated in the regulation of a variety of important genes, such as *RAS* and *HMGA2* (Johnson et al., 2005; Mayr et al., 2007). They found miRNA let-7f promotes β-catenin activity in hMSCs by repressing the translation of *Axin2* (Egea et al., 2012). One the other hand, in a study lately published, Kim et al. reported that upregulation of miR-205 in KB oral cancer cells could trigger cell apoptosis. Further mechanistic study identified *Axin2* as the direct target of miR-205 (Lee et al., 2013). They showed that over-expressed miR-205 in KB oral cancer cells suppresses *Axin2* expression through an interaction with its own binding site at *Axin2* 3′-UTR (64–92). However, it is unclear how reduced *Axin2* expression conferred by miR-205, rather than facilitates tumorigenesis as it usually does, contributes to the suppression of KB oral cancer cells.

## Epigenetic Modulations of Axin in Cancer

In additional to the post-transcriptional regulations mentioned above in affecting Axin levels, epigenetic modulations could also affect the expression of Axin and the change thereof may relate to tumor development. It is well accepted that genetic changes in components of Wnt signaling are closely related with human cancer. One leading example is colorectal cancer: alterations in Wnt-related genes, mainly APC, have been observed in ~90% of colorectal cancer (Miyaki et al., 1994; Najdi et al., 2011). In addition to genetic mutations, epigenetic modification is another common cause for altered gene expression, among which promoter methylation and histone modification are the most frequently observed. Axin is also frequently found mutated in cancer, contributing to the over-activation of Wnt signaling in these cancers, while studies regarding epigenetic alterations of Axin and its relationship to cancer remain relatively scant. As a negative regulator of Wnt signaling, tumor-specific promoter methylation or histone deacetylation will cause decreased Axin expression, which may prompt over-activation of Wnt signaling in cancer. Previously, epigenetic silencing of *Axin2* was reported in colorectal carcinoma with microsatellite instability (MSI+ CRC), and forced expression of *Axin2* results in rapid cell death in an MSI+ CRC cell line, indicating a role of epigenetic silencing of *Axin2* in carcinogenesis of MSI+ CRC (Koinuma et al., 2006). Lately, CpG islands of *Axin2* were found methylated in human neuroendocrine tumors (NETs) and Axin2 expression is correspondingly downregulated, which may contributes to the pathogenesis and growth of NETs (Kim et al., 2013a). A recent work carried out by Yang et al., also indicated that hypermethylated Axin gene may significantly correlate with the progression of lung cancer (Yang et al., 2013). On the other hand, Chen G. et al. reported that overexpression of Menin, a tumor suppressor protein mutated in patients with multiple endocrine neoplasia type 1, is associated with increased H3K4 trimethylation of the Axin2 gene promoter; while, inhibition of Menin expression by siRNA abrogates H3K4 trimethylation and gene expression of Axin2, indicating that Menin functions, at least partly, through affecting histone trimethylation of *Axin2* promoter (Chen et al., 2008). Recently, Histone deacetylase (HDAC) 1 and 2 are implicated in the regulation of Axin expression in non-small cell lung cancer (NSCLC) (Han et al., 2012). X-radiation and siRNA could inhibit expression of HDAC1 and HDAC2 and weaken its inhibitory effect on Axin, which upregulates Axin expression and induces apoptosis of lung cancer cells (Han et al., 2012).

## Concluding Remarks and Perspectives

Besides those post-transcriptional modulations mentioned above, another modification—methylation—is also observed for Axin (Cha et al., 2011; Huang et al., 2009). A recent work carried out by Cha et al. revealed that PRMT1 directly interacts with and methylates Axin, thus enhancing Axin stability (Cha et al., 2011; Huang et al., 2009). So far, our knowledge about post-transcriptional modifications of Axin is still very limited. More kinases, phosphatases, E3 ubiquitin ligases and probably other types of post-transcriptional regulations are expected to be identified in the near future to fit into the network that modulates Axin activity in canonical Wnt signaling. In light of current findings, Axin seems to be a flexible and dynamic molecule, which could undergo structural rearrangements for interacting with distinct partners. The three-dimensional structure of the full-length Axin will provide invaluable insights into the details regarding Axin auto-inhibition. On the other hand, increasing evidence indicates a role of Axin in nucleus (Nikuseva Martic et al., 2010; Pecina-Slaus et al., 2011; Schmitz et al., 2013; Willert and Jones, 2006). However, the precise role of nuclear Axin in Wnt signaling remains largely unknown, which warrants further investigation to facilitate our understanding of the function of nuclear Axin as well as its linkage to the cytoplasmic one. As an attractive therapeutic target, much effort has been made to identify chemical compounds modulating Axin activity. So far, most studies focus on small-molecules in improving Axin stability and thus attenuating Wnt signaling, among which inhibitors against Tankyrase are the best explored (Bao et al., 2012; Huang et al., 2009; James et al., 2012; Lu et al., 2009; Shultz et al., 2012). Tankyrase-mediated PARsylation of Axin stimulates Axin degradation via the ubiquitin-proteasome pathway (Huang et al., 2009). Thus, by inhibiting Tankyrase activity, Axin is spared from the consequent ubiquitination and degradation, which may antagonize Wnt signaling and inhibit tumor growth. Wnt/β-catenin signaling normally needs to be blocked for therapeutic purpose in cancer; however, it also plays an important role in hematopoietic stem cell (HSC) formation and self-renewal, and is required for HSC recovery after injury and in transplantation. Hence, our recent identification of HLY78 as a novel Wnt activator, may provide new chances for the circumstances where activation of Wnt signaling is desired, such as for the patients with bone marrow failure or undergoing post-HSC transplantation (Wang et al., 2013).

## Abbreviations

APC: adenomatous polyposis coli
GSK3: glycogen synthase kinase 3
HDAC: histone deacetylase
HSC: hematopoietic stem cell
NETs: neuroendocrine tumors
NSCLC: non-small cell lung cancer
Ub: ubiquitin
